# Obesity and obesity-associated cardiometabolic risk factors in indigenous Nenets women from the rural Nenets Autonomous Area and Russian women from Arkhangelsk city

**DOI:** 10.3402/ijch.v73.23859

**Published:** 2014-07-24

**Authors:** Natalia Petrenya, Magritt Brustad, Liliya Dobrodeeva, Fatima Bichkaeva, Gulnara Lutfalieva, Marie Cooper, Jon Øyvind Odland

**Affiliations:** 1 Department of Community Medicine, Faculty of Health Sciences, UiT The Arctic University of Norway, Tromsø, Norway; 2 Department of Community Medicine, Faculty of Health Sciences, Centre for Sami Health Research, UiT The Arctic University of Norway, Tromsø, Norway; 3 FGBUN Institute of Physiology of Natural Adaptations, Ural Branch, The Russian Academy of Sciences, Arkhangelsk, Russia; 4 Norwegian Institute of Food, Fisheries and Aquaculture Research, Tromsø, Norway

**Keywords:** obesity, women, cardiometabolic risk, Nenets, Russians, body mass index, waist circumference, waist-to-hip ratio, Arctic, ethnicity

## Abstract

**Background:**

The prevalence of obesity and obesity-related conditions varies by population groups. Indigenous women of the circumpolar north are believed to be at high risk of obesity.

**Objective:**

We studied, first the obesity prevalence in indigenous Arctic women, Nenets, compared to urban Russian women. Second, the association between obesity and cardiometabolic risk factors in the combined group of Nenets and Russian women. Third, ethnic differences in the association between obesity and cardiometabolic risk factors.

**Design:**

Cross-sectional study performed in 2008–2009. Subjects: 93 Nenets women, aged 19–77 (the indigenous village, the Nenets Autonomous Area) and 132 Russian women, aged 21–72 (Arkhangelsk city). Obesity was defined as body mass index (BMI)≥30 kg/m^2^, waist circumference (WC)≥88 cm and or waist-to-hip ratio (WHR)≥0.85%. We assessed associations between obesity and cardiometabolic risk factors by linear and logistic regression models that included covariates of ethnicity, age, smoking and physical activity. We also tested for interaction between obesity measurements and ethnicity.

**Results:**

Prevalence of obesity estimated through BMI, WC and WHR were 42.5, 45.3 and 41.9% in Nenets and 34.4, 46.4 and 29.5% in Russians, respectively, with no differences found. BMI, WC and WHR associated positively with triglycerides, fasting insulin and Homeostasis Model Assessment of Insulin Resistance index. In addition, BMI and WC correlated negatively with high-density lipoprotein cholesterol and positively with systolic blood pressure and apolipoprotein B/apoliporotein A–I ratio. WC explained significant variation in fasting glucose (FG) level. BMI predicted type 2 diabetes history. FG level associated strongly with ethnicity and was found to be higher in Russians.

**Conclusions:**

We found no differences in prevalence of obesity between Nenets and Russian females. Obesity was associated with cardiometabolic risk factors independently of ethnicity in the sample studied. There was no link between obesity measurements and ethnicity.

The World Health Organization (WHO) currently recognizes that overweight and obesity represent a rapidly growing threat to the health of populations (1). Obesity was found to be related to cardiometabolic risk factors. Severe chronic ailments, including type 2 diabetes mellitus (T2DM) and cardiovascular disease (CVD) are linked to obesity (2). Visceral adipose tissue accumulation has been found to be potentially hazardous (3).

Obesity affects all nations. Currently, ethnic minorities are believed to be at high risk. In Arctic populations, the mean body mass index (BMI), the prevalence of obesity, T2DM and its complications have increased rapidly in recent decades (4–7). The transition from traditional subsistence-oriented economies, causing a reduction in physical activity and the adoption of more Westernized nutritional habits among circumpolar groups are possible explanations for these negative health outcomes (8,9).

Increasing abdominal obesity in indigenous women is particularly alarming (10). Far North indigenous women often had higher mean BMI, mean waist circumference (WC), mean waist-to-hip ratio (WHR) and prevalence of general and central obesity when compared to women of different ethnic origin, but living in the same geographical areas (e.g. Sami women vs. Norwegian women) (11). Interestingly, the early study of cardiovascular risk factors in Sami women (the data collected in 1977–8) showed that Sami women were more obese but did not have a higher diabetes mellitus incidence than other women in the Finnmark County of Norway (12).

Cardiometabolic complications of obesity are somewhat puzzling issues among the Inuit. Indeed, a central fat deposition pattern and obesity have been observed often among the Inuit, especially women. Cardiometabolic risk factors have been found to be associated with obesity both in the Inuit and in other populations. However, several studies were consistent in observing that at each level of BMI or WC, the Inuit had more favourable profiles of cardiometabolic risk markers compared to Euro-Canadians and Danish participants (13–15). The unique diet and lifestyle in a cold climate environment, abdominal subcutaneous versus visceral fat deposition, genetics and/or other factors were suggested as contributing to differences in the impact of abdominal obesity on cardiometabolic risks (16). However, the evidence is sparse. The underlying mechanisms are incompletely understood and need to be addressed by further research.

According to the WHO statistics, in 2008, BMI≥30 kg/m^2^ (age-standardised estimate) in women in the Russian Federation was 29.8% (25.8–33.9%) (17). Few studies on obesity and cardiometabolic risks have been performed in high-latitude indigenous populations of the Russian Federation (18–21).

The present work focuses on females from a unique indigenous population of the Russian Arctic – Nenets. To the best of our knowledge, no systematic studies on obesity in relation to other cardiometabolic risk markers in the indigenous Arctic population of Nenets have been performed to date.

The main aims of the study were to investigate the differences in obesity prevalence in rural Nenets women and in urban Russian women as well as to estimate the association between obesity and cardiometabolic risk factors in the combined group of Nenets and Russian women. We also estimated ethnic differences in the association between obesity and cardiometabolic risk factors.

## Methods

### Ethics approval

The Ethical Committee at the Northern State Medical University, Arkhangelsk, approved the study. Written informed consent was obtained from each participant.

### Design and subjects

A cross-sectional study design was used. The study in Arkhangelsk city, in the North-West of Russia, was conducted between April 2008 and April 2009. In 2008, the total female population of Arkhangelsk city was estimated to be 193,485 (22). Fieldwork in the village of Nelmin-Nos (the Nenets Autonomous Area) was performed in February 2009. A sub-sample of ethnic Russian non-pregnant women free from type 1 diabetes from Arkhangelsk (132 women aged 21–72 years) and a sub-sample of ethnic Nenets non-pregnant women free from type 1 diabetes from the indigenous village of Nelmin-Nos (93 women aged 19–77 years) were used for this study. The total Nenets population of Nelmin-Nos was estimated to be 541 in 2009. There was a total of 273 eligible Nenets women aged 18 and older in the study village. The Nenets have inhabited the studied circumpolar area of the Eurasian continent for 1000s of years. The traditional economy of the Nenets was based mainly on herding, breeding reindeer, fishing and hunting. During recent decades, the traditional lifestyle of the Nenets people has been changing dramatically. Cardiovascular disease was reported to be a leading cause of death both in the rural Nenets Autonomous Okrug (683.0/100,000) and in the Arkhangelsk region (863.3/100,000) in 2008 (22).

### Recruitment and questionnaire survey

A similar recruitment strategy was implemented in the 2 study locations. Both verbal and written invitations to the health screening were distributed in several institutions in Arkhangelsk (e.g. libraries, retail outlets and cloths factory) and in public areas (e.g. medical centre, shops, school) in Nelmin-Nos. The screening consisted of a physical examination, blood sampling and a questionnaire survey. Our data from both locations were comparable and almost identical in methods.

Self-administration (44% women in Arkhangelsk and 49% women in Nelmin-Nos) and face-to-face interviews (56% women in Arkhangelsk and 51% women in Nelmin-Nos) were used in this study. The same questionnaire, written in Russian, was applied for both methods.

Four questions on ethnicity (including questions on the ethnicity of the participant's parents, ethnicity written in participant's documents and participant's self-definition) were developed based on a Russian validated version of the Survey of Living Conditions in the Arctic: Inuit, Sami, and the Indigenous Peoples of Chukotka study (SLiCA study) (23). The details are described elsewhere (24).

We collected information on self-reported diabetes (the type of diabetes was specified in the question), cardiovascular diseases, smoking habits and physical activity. Individuals were defined as having T2DM if they reported having T2DM, confirmed by a health professional and, ever having taken glucose lowering medication. Only one female with fasting glucose (FG) level≥7.0 mmol/L (7.22 mmol/L) and undiagnosed diabetes was detected. She was not defined as diabetic, based on 1 measurement of borderline glucose level. Women who reported daily smoking at the time of survey have been classified as smokers. Physical activity was categorized as sedentary, moderate and high based on a combined score of physical activity at work and during leisure time.

### Anthropometric and blood pressure measurements

Body weight in kg (±50 g) was measured with participants wearing light clothing using an electronic scale (A&D UC-322, Japan). Height was measured to the nearest 0.1 cm using a standard stadiometer. BMI was calculated as weight in kilograms divided by the square of height in metres.

Waist and hip circumference were measured in centimetres using a 1.5-m non-stretch tape. The WC was measured at the narrowest part between the lower rib and the iliac crest (the natural waist) or, in cases of indeterminate waist narrowing, halfway between the lower rib and the iliac crest. The measurements were recorded to the nearest 0.5 cm with the individual standing and breathing normally. The hip circumference was defined as the widest circumference over the buttocks. WHR was calculated as the WC divided by the hip circumference.

Systolic blood pressure (SBP) and diastolic blood pressure (DBP) were measured after 5 minutes rest in a seated position in standard measurement posture on the brachial artery with an OMRON M6 Comfort oscillometric automatic blood pressure monitor in the Arkhangelsk group and with standard sphygmomanometer using the auscultatory (manual) technique in the Nelmin-Nos group. The first reading was used for analysis.

### Laboratory measurements

All blood samples (both in Arkhangelsk and Nelmin-Nos) were taken after an overnight fast (at least 8 hours). Venous blood samples were collected using vacutainers and centrifuged within 30 minutes. Serum was aliquoted and stored frozen at −20°C. The samples from Nelmin-Nos were drawn by the same staff, stored frozen and transported to Arkhangelsk city. Measurements were performed at the laboratory of Biochemistry at the Institute of Environmental Physiology in Arkhangelsk with an automated clinical biochemical analyzer «MARS» (Infopia Co, Ltd, Anyang, Korea) or «Cary 50» spectrophotometer (Australia). Reagents from “Chronolab AG” (Switzerland) were used. Total cholesterol (TC), high-density lipoprotein cholesterol (HDL-C), triglyceride (TG) and FG were measured by enzymatic-colorimetric tests. Apolipoprotein A-I (ApoA-I) and apolipoprotein B (ApoB) were assayed by an imminoturbodimetric method with polyclonal goat serum anti-human apolipoprotein antibodies (“Chronolab AG”). The assay and calibrator concentration have been standardised against the WHO/IFCC SPI/0I standard for ApoA-I and the WHO/IFCC SP3/07 for ApoB (CDC, USA). Only 45 female samples in Arkhangelsk and 81 female samples in Nelmin-Nos were left for fasting insulin (FI) measurements. Insulin was measured by enzyme immunoassay with Evolis Fully Automated ELISA Processor, “Bio-Rad” (Germany) and kid «DRG» (Germany) (EIA-2935). Both external and internal quality controls were established. Measurements met the standards for the international Quality Assurance/Quality Control network. The analytic covariance for all parameters was ≤3%.

### Definitions

General obesity was estimated through BMI measurement. Cut-off value for general obesity was BMI ≥30 kg/m^2^. Central obesity was estimated through WC and WHR measurements. Cut-off value for WC was ≥88 cm and for WHR was ≥0.85.

The National Cholesterol Educational Program/Adult Treatment Panel III (NCE/ATP III) criteria were used to define metabolic syndrome (MetS). Any 3 of the following constituted a diagnosis: elevated WC (≥88 cm), elevated serum FG (≥6.1 mmol/L (or glucose lowering medication)), elevated TG (≥1.7 mmol/L (or cholesterol-lowering medication)), reduced HDL-C (<1.3 mmol/L (or cholesterol-lowering medication)) and elevated blood pressure (≥130 mmHg SBP and/or ≥85 mmHg DBP (or treatment for hypertension)). We used these criteria as the most practical instead of other alternatives. We also estimated reduced levels of HDL-C (<1.0 mmol/L in women) and elevated blood pressure (≥140 mmHg SBP and/or ≥90 mmHg DBP) based on a WHO definition (1998) (25,26).

Insulin resistance was defined by calculated Homeostasis Model Assessment of insulin resistance (HOMA-IR index). HOMA-IR index=FI (µU/mL)×FG (mmol/L)/22.5 (27).

### Statistical methods

If distribution was normal, continuous variables were presented as mean (standard deviation (SD)) and differences between residences were studied by unpaired t-test. If distribution was skewed, geometric means (GM) and 95% confidence interval (CI) were shown. In that case, we applied analysis of covariance (ANCOVA), where age was a single covariate. Categorical variables were presented as % from total number within residence. Differences were studied by Pearson Chi-Square test. The categorical variables were recoded as 0=Nenets, 1=Russians, 0=no diabetes, 1=a presence of diabetes, 0=non-smokers, 1=smokers. We created 2 dummy variables for physical activity pattern (the reference group was high level of physical activity). Variables SBP, TC, HDL-C, TG, ApoB/ApoA-I ratio, FG, FI and HOMA-IR were log10-transformed to correct for skewed distribution. Impact of obesity indexes (BMI, WC and WHR) and ethnicity on log10-transformed cardiometabolic risk factors (SBP, TC, HDL-C, TG, ApoB/ApoA-I ratio, FG, FI and HOMA-IR) was studied by linear regression analysis. Associations between obesity (BMI, WC and WHR), ethnicity and T2DM were estimated by logistic regression. We evaluated missing data issues. Little's Missing Completely at Random (MCAR) Test was used. The assumption that data are MCAR was met. We applied list-wise deletion for treatment of missing data. We adjusted all models for age, smoking and physical activity. Next, to assess whether there was an ethnicity-by-BMI (WC, WHR) difference, we tested for interaction between BMI (WC, WHR) by adding a multiplicative interaction term to the fully adjusted models.

A p-value of <0.05 (2-tailed tests) was defined as significant. A p-value of <0.10 for interaction term was defined as significant. Statistical analysis was performed, using SPSS for Windows statistical package (version 19.0; SPSS Inc. Chicago, IL, USA).

## Results

### Anthropometric data and characteristics of the samples

Characteristics of the study participants are summarized in Table I.

**Table I:** Characteristics of women by residence (n=225)^a^

Characteristics	Arkhangelsk, N=132	Nelmin-Nos, N=93	p
Age (years), median (IQR)	51 (38–55)	46 (32–53)	0.013^b^
Height (cm), mean (SD)	161.6 (5.8)	151.9 (6.7)	<0.001^c^
Weight (kg), GM (95% CI)	71.3 (68.7–74.0)	64.9 (61.9–67.8)	0.001^d^
WC (cm), GM (95% CI)	85.5 (83.4–87.9)	85.7 (82.8–88.5)	0.964^d^
Hip circumference (cm), GM (95% CI)	106.2 (104.2–108.1)	102.3 (100.0–104.7)	0.014^d^
BMI (kg/m^2^), GM (95% CI)	27.2 (26.2–28.1)	28.6 (27.2–29.6)	0.126^d^
Waist-to-hip circumference ratio (%), GM (95% CI)	0.81 (0.79–0.82)	0.84 (0.82–0.85)	0.001^d^
BMI groups (kg/m^2^), n (%)			
≤29.9	80 (65.6)	46 (57.5)	
≥30	42 (34.4)	34 (42.5)	0.247^e^
WC groups (cm), n (%)			
≤87.9	60 (53.6)	41 (54.7)	
≥88	52 (46.4)	34 (45.3)	0.883^e^
WHR groups, n (%)			
≤0.84	79 (70.5)	43 (58.1)	
≥0.85	33 (29.5)	31 (41.9)	0.081^e^
TC≥5.1 mmol/L, n (%)	88 (68.8)	45 (48.9)	0.003^e^
HDL-C<1.3 mmol/L, n (%)	84 (68.3)	59 (64.1)	0.522^e^
HDL-C<1.0 mmol/L, n (%)	37 (30.1)	24 (39.3)	0.520^e^
TG≥1.7 mmol/L, n (%)	26 (20.3)	13 (14.1)	0.236^e^
Apo B/A-I ratio (%), GM (95% CI)	1.0 (0.93–1.08)	0.92 (0.85–1.01)	0.175^d^
FG (mmol/L), GM (95% CI)	4.9 (4.8–5.1)	4.3 (4.2–4.5)	<0.001^d^
FG≥6.1 mmol/L, n (%)	6 (4.7)	5 (5.4)	0.802^e^
FI (µU/ml), GM (95% CI)	7.3 (5.8–9.1)	6.8 (5.9–8.0)	0.623^d^
HOMA-IR, GM (95% CI)	1.5 (1.2–2.0)	1.3 (1.0–1.5)	0.190^d^
SBP (mmHg), GM (95% CI)	124.2 (120.8–127.6)	135.8 (131.8–140.0)	<0.001^d^
DBP (mmHg), GM (95% CI)	84.0 (82.0–86.0)	82.4 (80.4–84.5)	0.281^d^
SBP≥140 mmHg or diastolic blood pressure≥90 mmHg, n (%)	41 (39.0)	38 (44.2)	0.473^e^
SBP≥130 mmHg or diastolic blood pressure≥85 mmHg (or treatment for hypertension), n (%)	54 (51.4)	46 (53.5)	0.777^e^
MetS, n (%)	34 (35.1)	21 (30.4)	0.533^e^
Diabetes mellitus, n (%)	6 (5.1)	5 (5.9)	0.808^e^
Angina, myocardial infarction or stroke, n (%)	14 (10.8)	8 (8.8)	0.629^e^
Current smoker, n (%)	16 (13.3)	13 (14.0)	0.892^e^
Physical activity, n (%)			
Low	35.0 (43)	42.6 (29)	0.575^e^
Moderate	46.3 (57)	41.2 (28)	
High	18.7 (23)	16.2 (11)	

IQR=interquartile range; GM=geometric mean; WC=waist circumference; BMI=body mass index; TC=total cholesterol; HDL-C=high-density lipoprotein cholesterol; TG=triglycerides; SD=standard deviation; CI=confidence interval; Apo=apolipoprotein; FG=fasting glucose; HOMA-IR=Homeostasis Model Assessment of insulin resistance; SBP=systolic blood pressure; FI=fasting insulin; DBP=diastolic blood pressure; MetS=metabolic syndrome; WHR=waist-to-hip-ratio.

a N within the groups may not be total 225 due to missing values.

b p-values are obtained by Mann–Whitney test.

c p-value is obtained by independent t-test.

d p-values are obtained by ANCOVA, age adjusted.

e p-values are obtained by Pearson Chi-Square test.

Russian women were older than Nenets women (median 51 years vs. 46 years respectively, p=0.013). Anthropometric characteristics were considerably different between the groups. Women of Nenets ethnicity were approximately 10 cm shorter (p<0.001) and had lower body weight (p=0.001). Hip circumference was smaller in Nenets women (p=0.014), but WC was not different (86 cm in both residences). Therefore, the calculated WHR was greater (p=0.001) in Nenets participants. The percentage of women with general obesity was 34.4% in Russian women and 42.5% in Nenets women (p=0.247). The percentage of women with central obesity estimated through WC (p=0.883) and WHR (p=0.081) was not different across residences and comprised 46.4 and 29.5% in urban Russians and 45.3 and 41.9% in rural Nenets. The prevalence of MetS was high in Nenets women and equal to the prevalence found in the urban Russian women (30.4% vs. 35.1%, respectively, p=0.533). Low HDL-C, high blood pressure and increased WC were frequent in both residences with no differences between the populations. Nenets women participants were not involved substantially in physical labour. They worked predominantly at school, kindergartens, medical centres, shops and so on (data are not shown).

### Association between general and central obesity measurements and cardiometabolic risk markers

The relationships of BMI, WC and WHR with cardiometabolic risk factors are shown in Table II. BMI, WC and WHR associated positively with TG, FI and HOMA IR-index. In addition, BMI and WC correlated negatively with HDL-C and positively with SBP and Apo B/Apo A-I ratio. Only WC associated positively with FG level. BMI associated positively with a history of self-reported T2DM (B=−0.185, SE=0.091, p=0.042, Odds Ratio=0.831, 95% CI Odds Ratio=0.695, 0.994).

**Table II:** Impact of obesity indexes and ethnicity^a^ on log10-transformed cardiometabolic risk factors in combined sample of women

	Models with BMI and ethnicity as independent variables				Models with WC and ethnicity as independent variables				Models with WHR and ethnicity as independent variables			
	
	BMI		Ethnicity		WC		Ethnicity		WHR		Ethnicity	
	
	B (CI 95%)	p	B (CI 95%)	p	B (CI 95%)	p	B (CI 95%)	p	B (CI 95%)	p	B (CI 95%)	p
SBP	0.003 (0.001, 0.005)	0.001	−0.031 (−0.05, −0.012)	0.002	0.001 (0.000, 0.002)	0.005	−0.038 (−0.058, −0.018)	<0.001	0.101 (−0.082, 0.284)	0.278	−0.031 (−0.052, −0.010)	0.004
TC	−0.002 (−0.005, 0.001)	0.138	0.047 (0.017, 0.077)	0.002	−0.001 (−0.002, 0.000)	0.137	0.046 (0.015, 0.078)	0.004	−0.124 (−0.404, 0.155)	0.380	0.039 (0.006, 0.071)	0.021
HDL-C	−0.005 (−0.009, −0.001)	0.019	−0.014 (−0.056, 0.027)	0.498	−0.003 (−0.004, −0.001)	0.006	−0.014 (−0.057, 0.029)	0.518	−0.274 (−0.664, 0.115)	0.166	−0.027 (−0.072, 0.018)	0.237
TG	0.010 (0.004, 0.016)	0.001	0.061 (−0.003, 0.125)	0.061	0.005 (0.002, 0.008)	<0.001	0.041 (−0.026, 0.108)	0.231	1.046 (0.454, 1.638)	0.001	0.077 (0.007, 0.146)	0.031
Apo B/Apo A–I	0.006 (0.001, 0.011)	0.029	0.049 (−0.007, 0.105)	0.087	0.003 (0.000, 0.005)	0.028	0.044 (−0.014, 0.102)	0.137	0.297 (−0.216, 0.810)	0.255	0.051 (−0.009, 0.111)	0.096
FG	0.001 (−0.001, 0.004)	0.186	0.070 (0.047, 0.094)	<0.001	0.001 (0.000, 0.002)	0.040	0.067 (0.043, 0.091)	<0.001	0.185 (−0.028, 0.399)	0.088	0.075 (0.050, 0.100)	<0.001
FI	0.026 (0.015, 0.038)	<0.001	0.173 (0.053, 0.294)	0.005	0.012 (0.006, 0.017)	<0.001	0.127 (−0.004, 0.258)	0.058	1.839 (0.761, 2.916)	0.001	0.190 (0.054, 0.327)	0.007
HOMA–IR	0.029 (0.016, 0.042)	<0.001	0.240 (0.108, 0.372)	0.001	0.013 (0.007, 0.019)	<0.001	0.185 (0.040, 0.329)	0.013	2.079 (0.899, 3.259)	0.001	0.255 (0.106, 0.405)	0.001

BMI=body mass index; WC=waist circumference; WHR=waist-to-hip ratio; CI=confidence interval; SBP=systolic blood pressure; TC=total cholesterol; HDL-C=high density lipoprotein cholesterol; TG=triglycerides; Apo=apolipoprotein; FG=fasting glucose; FI=fasting insulin; HOMA-IR index=Homeostasis Model Assessment of insulin resistance.

a All the models included covariates age, smoking and physical activity.

### Association between ethnicity and cardiometabolic risk markers, the effect of ethnicity on obesity-associated risk

The associations between ethnicity and cardiometabolic risk factors, estimated by multiple linear regressions are shown in Table II. In the models with BMI Russian versus Nenets, ethnicity was positively associated with TC, FG, FI and HOMA-IR levels and negatively with SBP level. In the models with WC Russian versus Nenets, ethnicity was positively associated with TC, FG and HOMA-IR levels and negatively with SBP level. In the models with WHR Russian versus Nenets, ethnicity was positively associated with TC, TG, FG, FI and HOMA-IR levels and negatively with SBP level. In the logistic regression model, ethnicity had no impact on T2DM history (p=0.335).

The test for interaction (BMI*ethnicity, WC*ethnicity, WHR*ethnicity) provided no evidence for differential associations across ethnic groups. For log 10-transformed TC levels, we found significant interactions only for BMI*ethnicity (B (95% CI)=−0.004 (−0.009, 0.001), p=0.098), WC*ethnicity (B (95% CI)=−0.003 (−0.005, 0.000), p=0.023), WHR*ethnicity (B (95% CI)=−0.558 (−1.018, −0.098), p=0.018). However, obesity indexes in the regression models had no impact on TC level in combined samples (Table II) as in samples, stratified by ethnicity (data are not shown).

## Discussion

The present study has demonstrated that first, there were no differences in the prevalence of general and central obesity between Nenets and Russian females and second, anthropometric indexes of general and central obesity explained significant variation in cardiometabolic risk factors independently of ethnicity, age, smoking and physical activity in the combined group of Nenets and Russian women. Although ethnicity had an impact on cardiometabolic risk factors, the associations between obesity and cardiovascular risk factors appeared not to be different between participants of Russian and Nenets origin.

Until these data become available, the obesity rate among Nenets women using general WHO cut-offs is considered high. Our data on prevalence of obesity are in line with previous studies of Inuit women (28,29). The cross-sectional International Polar Year Inuit Health Survey for adults in 2007–2008 showed that 41.6% of Canadian Inuit women had a BMI ≥30 kg/m^2^ (30). Our results from the urban Russian women are comparable with WHO statistics on the prevalence of BMI ≥30 kg/m^2^ in Russian women reported in 2008 (17).

The strongest associations were found between adiposity indexes and TG level, FI level and HOMA-IR index. In addition, individuals with higher BMI had a greater chance of having T2DM. A large WC was associated with high FG concentrations. Almost no information is available on the prevalence of MetS, glucose intolerance and T2DM in indigenous populations of Russia. The early study by Dogadin et al. reported low prevalence of T2DM in indigenous peoples of northern areas of the Krasnoyarsk region of Siberia (the age-standardised T2DM prevalence was 2.5/1,000 (95% CI: 1.5–3.6)). In that study, no cases of glucose intolerance were found among 596 Evenks, the indigenous population of Siberia (20). The prevalence of diabetes was reported to be 1.8/1,000 among the Chukchi and Eskimo of Chukotka (21). One further study, conducted on the indigenous Siberian population (Yakut), reported FG (mean 4.46 mmol/L in females) and MetS (8% in females) at relatively low levels. However, the authors expected future increases in MetS and impaired FG in this population due to rising rates of obesity (19). In line with this study, we found that the FG level was considerably lower in Nenets women, when compared to Russian women (4.3 vs. 4.9 mmol/L). On the contrary, the rate of MetS was high in Nenets women (30.4%). According to the information provided by medical staff of the village, where we collected the samples, only 3 cases of diabetes were registered in the total Nelmin-Nos population in 1994 while this number had increased to 12 by 2008. Improved diagnostics as well as an increasing diabetes rate could possibly account for that.

Height, weight and hip circumference were considerably lower in Nenets women. It has previously been reported that Arctic indigenous people (Inuit and Far East Asians) have shorter legs and relatively higher sitting heights compared with all other populations studied (31). No specific guidelines with respect to recommended anthropometric characteristics exist for the Nenets population. Consequently, using BMI values to estimate cardiometabolic risk in Nenets women may overestimate the number of individuals that are overweight and obese when general WHO criteria are used. Anthropometric indexes cannot provide information on body fat quantities and visceral vs. subcutaneous fat distribution. The fat distribution may also be different between Nenets and Russian females. The reference methods, for example, computer tomography (CT) or magnetic resonance imaging, may provide this information. However, the use of these methods in epidemiological research is very limited (equipment is extremely expensive and stationary; in CT ionizing radiation is used). To investigate whether general BMI, WC and WHR cut-offs are applicable for the Nenets population, more complex systematic studies, using techniques validated against the reference methods are needed.

A limitation of the study was the relatively small sample size. We had difficulties in obtaining a representative sample. This could reduce the external validity and generalizability of our findings. Only one settlement was studied in the rural Nenets Autonomous Area. To draw firm conclusion about the Nenets population of the region in general, more expanded and representative studies, including participants from several locations, are necessary. Young women were to some degree underrepresented in the present study. The “healthy worker” effect might have taken place as well. It is likely that older Nenets women with pre-existing health problems were more likely to participate in the study and this resulted in an overestimated prevalence of obesity, MetS and T2DM among the adult female Nelmin-Nos population. However, a similar selection bias could have taken place in the Russian group as well. That's why we believe that comparison between the ethnic groups was not greatly affected. One more limitation is that 2 different methods, face-to-face interviews and self-administration of questionnaires, were used. This may lead to information bias. It is recommended that 3 consecutive measurements of arterial blood pressure should be performed and their mean (or the mean of the second and third measurement) used in the analyses. We used only one reading of blood pressure, which may be less accurate. In our study we used the HOMA-IR index, which is less accurate then, for example. euglycemic clamp, the reference method for estimating insulin resistance. We were unable to adjust for some possible confounders. For example, alcohol consumption might have influenced our results. The data on alcohol consumption (the amount of alcohol units a day and CAGE test) are of a low quality and with a high number of missing values. We have not analysed postprandial hyperglycaemia in our study. Postprandial hyperglycaemia has been shown to induce vascular endothelial dysfunction and to better predict CVD risk and mortality than FG (32).

The strengths of our study is that first of all the unique, remote not readily-accessible for research ethnically homogenous Nenets population (the majority of the indigenous village participants had both ethnic Nenets for parents) was studied. Ethnicity was clearly defined by the questionnaire survey. There was a good comparability of the data from these 2 completely different locations, because a single study protocol was used. In addition, the same team of qualified technicians, medical doctors and scientists worked both in Arkhangelsk and Nelmin-Nos. It is important for comparison that all the samples were drawn in the morning after an overnight fast, analysed in the same laboratory using standardised laboratory techniques and kits. Moreover, in our study the cardiometabolic risk factors included not routinely used ApoA-I and ApoB measurements, which are among the best predictors of CVD risk.

## Conclusion

We found that obesity in women is a concern and adiposity was clearly associated with cardiometabolic risk factors independent of ethnicity in the group comprising urban Russian and rural Nenets female populations. It is, however, possible that the prevalence of obesity, MetS and T2DM among the Nenets women were overestimated. A large representative longitudinal study using comprehensive anthropometric techniques is required to confirm that the prevalence of obesity is high and is increasing among the Nenets women.
